# LncRNA H19 interacted with miR‐130a‐3p and miR‐17‐5p to modify radio‐resistance and chemo‐sensitivity of cardiac carcinoma cells

**DOI:** 10.1002/cam4.1860

**Published:** 2019-03-06

**Authors:** Jianguang Jia, Xinxin Zhang, Dankai Zhan, Jing Li, Zhixiang Li, Hongbo Li, Jun Qian

**Affiliations:** ^1^ Department of Surgical Oncology The First Affiliated Hospital of Bengbu Medical College Bengbu China

**Keywords:** cardiac cancer, cell apoptosis, cell viability, chemo‐sensitivity, lncRNA H19, miR‐130a‐3p, miR‐17‐5p, radio‐sensitivity

## Abstract

The current investigation explored the synthetic contribution of lncRNA H19, miR‐130a‐3p, and miR‐17‐5p to radio‐resistance and chemo‐sensitivity of cardiac cancer cells. Totally 284 human cardiac cancer tissues were gathered, and they have been pathologically diagnosed. The cardiac cancer cells were isolated with utilization of the mechanic method. Moreover, cisplatin, adriamycin, mitomycin, and 5‐fluorouracil were designated as the chemotherapies, and single‐dose X‐rays were managed as the radiotherapy for cardiac cancer cells. We also performed luciferase reporter gene assay to verify the targeted relationship between H19 and miR‐130a‐3p, as well as between H19 and miR‐17‐5p. Finally, mice models were established to examine the functions of H19, miR‐130a‐3p, and miR‐17‐5p on the development of cardiac cancer. The study results indicated that H19, miR‐130a‐3p, and miR‐17‐5p expressions within cardiac cancer tissues were significantly beyond those within adjacent nontumor tissues (*P* < 0.05), and H19 expression was positively correlated with both miR‐130a‐3p (*r*_s_ = 0.43) and miR‐17‐5p (*r*_s_ = 0.49) expressions. The half maximal inhibitory concentrations (IC50) of cisplatin, adriamycin, mitomycin, and 5‐fluorouracil for cardiac cancer cells were, respectively, determined as 2.01 μg/mL, 8.35 μg/mL, 24.44 μg/mL, and 166.42 μg/mL. The overexpressed H19, miR‐130a‐3p, and miR‐17‐5p appeared to improve the survival rate and viability of cardiac cancer cells that were exposed to chemotherapies and X‐rays (all *P* < 0.05). It was also drawn from luciferase reporter gene assay that H19 could directly target miR‐130a‐3p and miR‐17‐5p, thereby modifying the sensitivity of cardiac cancer cells to drugs and X‐rays (*P* < 0.05). Finally, the mice models also produced larger tumor size and higher tumor weight, when H19, miR‐130a‐3p, or miR‐17‐5p expressions were up‐regulated within them (*P* < 0.05). In conclusion, H19 could act on miR‐130a‐3p or miR‐17‐5p to alter the radio‐ and chemo‐sensitivities of cardiac cancer cells, helping to improve the radio‐/chemotherapies for cardiac cancer.

## 1 INTRODUCTION

Gastric cancer is one of the most commonly diagnosed cancers around the world, and its incidence in China accounts for about 24% of the world.^1^ Cardiac cancer, a subtype of gastric cancer, occurs 5 cm above and below the junction of esophagus and stomach. Its symptoms are reflected as discomfort in upper abdominal, fullness after light meals, indigestion, and pain in precordium. Although surgeries, radiotherapies, chemotherapies, and immunotherapies have been adopted to treat cardiac cancer, the 5‐year survival rate of cardiac cancer patients still remained poor at 24%.^2^ Hence, defining the mechanisms inherent in cardiac cancer could assist in perfecting treatment efficacy for cardiac cancer patients.

LncRNAs, a class of noncoding RNAs with a length >200 bp, were identified as potent candidates in regulating tumor etiology,^3^, ^4^, ^5^, ^6^ for that they cast a dominant role in epigenetic, transcriptional, and posttranscriptional regulations within neoplasms.^7^ Considering that cardiac cancer was a branch of gastric cancer, lncRNAs affecting gastric cancer development were deemed as probable contributors to cardiac cancer. Of note, lncRNA H19 (NR_002196, 2300 bp, Ensembl: ENSG00000130600), which encoded a 2.3 kb‐long imprinted lncRNA of maternal origin, was significantly more expressed within primary gastric cancer tissues than within normal stomach tissues.^8^ This lncRNA also geared up proliferation, migration, and invasion of gastric cancer cells through mediating RUNX1 or Akt/mTOR signaling.^9^, ^10^, ^11^ Apart from gastric cancer, H19 was highly expressed within esophageal cancer, bladder cancer, breast cancer, gastric cancer, and colon cancer as well.^12^, ^13^, ^14^, ^15^, ^16^ Nonetheless, knockout of H19 within animal models of colorectal cancer generated incremental polyps, implying that the function of H19 might be disparate for manifold disorders.^17^ Furthermore, facilitating tumor progression could enable less sensitivity of tumor cells to chemo‐ and radiotherapies, for example, underexpressed H19 promoted the susceptibility of gastric cancer cells to docetaxel.^18^ Nevertheless, hardly any investigations have been performed to make certain the effects of H19 on chemo‐ and radio‐ sensitivities of cardiac cancer cells.

Moreover, lncRNAs interplaying with miRNAs have also been confirmed to participate in modifying cancer progression by activating or inactivating genetic expressions.^19^ For instance, it was manifested that H19 could accelerate onset of gastric cancer, colorectal cancer, and glioma by interacting with miR‐675.^10^, ^20^, ^21^ The Starbase 2.0 software^22^ also provided predictions about the targeted miRNAs of H19, among which miR‐130a‐3p (NR_029673, 22 nt, miRbase accession number: MIMAT0000425) and miR‐17‐5p (NR_029487, 23 nt, miRbase accession number: MIMAT0000070) were outstanding biomarkers for the development of gastric cancer.^23^, ^24^ The miR‐130a‐3p therein deteriorated the development of hepatocellular carcinoma (HCC), and overexpression of miR‐130a led to incremental resistance of HCC cells to cisplatin.^25^, ^26^ In regard to miR‐17‐5p, it negatively regulated p21 and TP53INP1 to facilitate migration of gastric cancer cells,^24^ and its expression was obviously up‐regulated within paclitaxel‐resistant lung cancer cells than within paclitaxel‐sensible lung cancer cells.^27^ Despite that miR‐130a‐3p and miR‐17‐5p participated in modifying cancer development and chemo‐/radio‐ sensitivities of tumor cells, what molecules mastered their function and what mechanisms they mediated to change cardiac cancer cells’ chemo‐ or radio‐resistance remained ambiguous.

Hence, it was hypothesized that H19 and miR‐130a‐3p or miR‐17‐5p could synthetically contribute to differed radio‐ and chemo‐sensitivity of cardiac cancer cells. Thus, this investigation was aimed to partly unveil this potential mechanism on the basis of in‐vitro and in‐vivo experiments, which could help to guide radio and chemotherapies for cardiac cancer.

## 2 MATERIAL AND METHODS

### 2.1 Collection of cardiac cancer tissues

Two hundred and eighty‐four cardiac cancer tissues and corresponding adjacent normal tissues were taken from the First Affiliated Hospital of Bengbu Medical College. The time span stretched from March 2013 to June 2017. The patients were all pathologically diagnosed as cardiac cancer, and they did not perform chemotherapy or radiotherapy preoperatively. All the patients have signed informed consents, and this study was approved by the First Affiliated Hospital of Bengbu Medical College and the Ethics Committee of the First Affiliated Hospital of Bengbu Medical College.

### 2.2 Separation and culture of cardiac cancer cells

Under aseptic conditions, human cardia cancer tissues were removed from one patient, and single‐cell suspension of tumor cells was obtained utilizing mechanical method. We also purchased normal gastric mucosal cell line (ie, GES‐1) from American Type Culture Collection (ATCC). The cells were cultured within DMEM (HyClone, South Logan, UT, USA) that were added with 10% FBS (Life Technologies, Gaithersburg, MD, USA), 0.1 mmol/mL sodium pyruvate (Gibco, Grand Island, NY, USA), 50 U/mL penicillin, and 50 μg/mL streptomycin, and they were ensured in 5% CO_2_ and 90% humidity at 37°C.

### 2.3 Chemo‐therapeutics

Single‐cell suspension was added to the 96‐well plates at the density of 5 × 10^3^, and they were supplemented with cisplatin, adriamycin, mitomycin, and 5‐fluorouracil. The control group was managed to be without the addition of drugs. After being placed in CO_2_ incubator at 37°C for 48 hours, 20 μL MTT (5 mg/mL, Sigma, St. Louis, MO, USA) was supplemented into each well. The wells were cultured for 2 hours before microplate reader (Bio‐Rad, Hercules, CA, USA) was adopted to detect the absorbance values (A) at the wavelength of 570 nm.

### 2.4 Radio‐therapeutics

The cell lines at the density of 3 × 10^5^/well were divided into the unirradiated control, 2, 4, 6, and 8 Gy groups. They received one‐fractional irradiation from linear accelerator (Siemens, Princeton, NJ, USA), and 6 MeVX ray was emitted 100 cm distant from source skin at the absorbed‐dose rate of 2 Gy/min. After postirradiation culture for 24 hours, 0.25% trypsin was prepared to digest the cells, and the cell suspension was again cultivated in 5% CO_2_ and saturated humidity at 37°C for 12 days. The culture would be terminated if visible colonies were found, and the supernatants were discarded before twice of rinse with PBS. Subsequently, crystal violet staining solution (Sangon Biotech, Shanghai, China) was applied for 15‐minutes fixation, and then the fixation fluid was gently washed off prior to air‐drying. The plates were reversely placed, and one transparent adhesive film was superimposed. The colonies with at least 50 cells were counted under the microscope, and the survival fraction (SF) of cells was calculated.

### 2.5 Cell transfection

The pcDNA3‐H19 and pcDNA3 were provided by Invitrogen (Carlsbad, CA, USA), and si‐H19 and si‐NC were synthesized by Ribobio (Guangzhou, China) based on siDirect software (https://maidesigner.invitrogen.com/rnaexpress; Table S1). Moreover, the miR‐130a‐3p mimic, miR‐130a‐3p inhibitor, miR‐17‐5p mimic, miR‐17‐5p inhibitor, and miR‐NC were purchased from Genepharma (Shanghai China). The transfections of plasmid and oligonucleotide were performed utilizing Lipofectamine 2000 reagent (Life Technologies). After transfection for 48‐72 hours, the cells were harvested for further experiments.^28^

### 2.6 Cell apoptosis assay

Cells were double‐stained in line with the guidelines of Annexin V‐FITC Apoptosis Assay Kit (Vazyme, Nanjing, China). The apoptotic conditions of the cells were analyzed with assistance of FACS Calibur flow cytometer (BD Biosciences, San Jose, CA, USA), resulting in a bivariate scattergram that presented viable cells labeled by FITC‐/PI‐ in the lower‐left quadrant and FITC‐/PI+ in the upper‐left quadrant, necrotic cells labeled by FITC+/PI+ in the upper‐right quadrant, as well as early‐apoptotic cells labeled by FITC+/PI‐ in the lower‐right quadrant.

### 2.7 Colony formation assay

The trypsinized cells were added into 6‐well plates at the density of 3000/well. After 9‐day regular culture in 5% CO_2_ at 37°C, cells were fixed with methanol for 20 minutes, and crystal violet (Sigma) was applied for staining. The colonies that included >50 cells were counted under the microscope.

### 2.8 Quantitative reverse transcription‐polymerase chain reaction (qRT‐PCR)

TRIzol reagent (Invitrogen) was employed to extract total RNA from cardiac cancer tissues or cells, which were then reversely transcribed (Invitrogen). Based on primers (Table 1) designed with ABI Primer Express software and synthesized by Shanghai Sangon (China), PCR was performed with SYBR‐green PCR Master Mix within Fast Real‐time PCR 7500 system (Applied Biosystems, Foster City, CA, USA). The PCR conditions of H19 were specified as: (a) predenaturation at 95°C for 30 seconds, (b) 38 cycles of denaturation at 95°C for 5 seconds and annealing at 60°C for 30 seconds, and (c) extension at 40°C for 5 minutes. As for miR‐130a‐3p and miR‐17‐5p, their PCR, conditions were particularized as: (a) predenaturation at 95°C for 30 seconds, (b) 40 cycles of denaturation at 95°C for 4 seconds and annealing at 56°C for 20 seconds, and (c) extension for 5 minutes. GAPDH was adopted as the internal reference for H19, and U6 snRNA was set as the internal reference for miR‐130a‐3p and miR‐17‐5p. The relative expressions of target genes were calculated following the 2^−∆∆^ *^Ct^* method.

**Table 1:** List of primers utilized for quantitative real‐time polymerase chain reaction (qRT‐PCR)

Gene	Primer sequence
Lnc RNA H19	5ʹ‐ATCGGTGCCTCAGCGTTCGG‐3ʹ (forward)
	5ʹ‐CTGTCCTCGCCGTCACACCG‐3ʹ (reverse)
GAPDH	5ʹ‐ACCTGACCTGCCGTCTAGAA‐3ʹ (forward)
	5ʹ‐TCCACCACCCTGTTGCTGTA‐3ʹ (reverse)
miR‐130a‐3p	5ʹ‐TTGCGATTCTGTTTTGTGCT‐3ʹ (forward)
	5ʹ‐GTGGGGTCCTCAGTGGG‐3ʹ (reverse)
miR‐17‐5p	5ʹ‐CCAGGATCCTTTATAGTTGTTAGAGTTTG‐3ʹ (forward)
	5ʹ‐CGGAATTCTAATCTACTTCACTATCTGCAC‐3ʹ (reverse)
U6	5ʹ‐GCTTCGGCAGCACATA‐3ʹ (forward)
	5ʹ‐ATGGAACGCTTCACGA‐3ʹ (reverse)

### 2.9 Western blotting

RIPA lysis buffer (Sigma, USA) supplemented with proteinase inhibitor (Roche Applied Science, Indianapolis, IN, USA) was managed to extract total protein, whose concentration was determined via BCA kit (Thermo Fisher Scientific, Waltham, MA, USA). Exactly 30 μg protein extracts were electrophoresed within 6%‐10% polyacrylamide gel and were then transferred onto the PVDF membrane. Subsequently, the membranes were blocked at room temperature for 1 hour, and we added rabbit anti‐human monoclonal antibodies for E‐cadherin, vimentin, and N‐cadherin (1:1000, Proteintech, Manchester, UK), along with rabbit anti‐human GAPDH monoclonal antibody (1:1000, CST, Danvers, MA, USA). After that, corresponding mouse anti‐rabbit secondary antibodies for E‐cadherin, vimentin, and N‐cadherin (1:10 000, CST) were supplemented for 1‐hour culture at room temperature. Finally, ECL detection reagent (Millipore, Billerica, MA, USA) was applied for development, and images were analyzed with Gene Genius gel imaging system (Syngene, Cambridge, UK).

### 2.10 Dual luciferase reporter gene assay

The chemically synthesized H19 fragments that included predicted miR‐130a‐3p‐binding sites were cloned into the Xhol and Notl sites of pGL3 vector (Promega, Madison, WI, USA), through which H19‐Wt was obtained. Correspondingly, H19‐Mut1 for miR‐130a‐3p was constructed similarly, except that the hsa‐miR‐130a‐3p‐binding sites were mutated. Abiding by the same logic, H19‐Mut2 for miR‐17‐5p was accomplished. The transfection procedures were followed exactly as the reference manuals. Firstly, 50 μL Opti‐MEM was poured to dilute 0.8 μg reporter vectors, 0.02 μg pRL‐SV40 plasmid control vector, and 20 pmol RNA segment. The cells were randomly divided into H19‐Wt+miR‐130a‐3p mimic, H19‐Wt+miR‐NC, H19‐Mut1+miR‐130a‐3p mimic, H19‐Mut1+miR‐NC, H19‐ Mut2+miR‐17‐5p mimic, and H19‐Mut2+miR‐NC groups. After standing at room temperature for 5 minutes, 2 μL Lipofectamine^™^ 2000 (Invitrogen) that was diluted by 50 μL Opti‐MEM was, respectively, mixed with plasmids and RNA segments. After gently blending them and making them stand for 20 minutes, the groups were added into 24‐well plates and were cultured at 37°C for 6 hours. The DMEM was replaced, and cells were cultured for another 48 hours. After evenly mixing 20 μL cell lysis buffer with 100 μL luciferase assay reagent II (LARII), we used fluorescence spectrometer (Promega) to monitor firefly luciferase activity, and then added 100 μL hemo‐fluorescein reagent to detect the activity of renilla luciferase. The ratio of firefly luciferase activity and renilla luciferase was calculated as the relative activity values for the following statistical analyses.

### 2.11 Establishment of cardiac cancer mice models and detection of tumor size

The BALB/C nude mice (n = 100) at the age of 4‐6 weeks old were collected from the experimental animal center of the First Affiliated Hospital of Bengbu Medical College. The mice were averagely divided into NC (n = 10), miR‐NC (n = 10, pcDNA (n = 10), pcDNA‐H19 (n = 10), si‐H19 (n = 10), miR‐130a‐3p mimic (n = 10), miR‐130a‐3p inhibitor (n = 10), miR‐17‐5p mimic (n = 10), miR‐17‐5p inhibitor (n = 10), and miR‐130a‐3p mimic+miR‐17‐5p mimic (n = 10) groups. We subcutaneously injected 200 μL cardiac cancer cell solution (5 × 10^6^/100 μL) into the oxter or back of each rat. After 5‐6 weeks, obvious projections were found, and the mice were sacrificed after being intraperitoneally injected with pentobarbital sodium (100 mg/kg). Then, the removed tumors were weighed, and their volumes were calculated as per the formula: (*D* × *d*2 × *π*)/6 (*D*: the longer diameter; *d*, the shorter diameter). The procedures mentioned above were performed in accordance with guidelines within the Institutional Animal Care and Use Committee of Bengbu Medical College.

### 2.12 Statistical analyses

The statistical SPSS 17.0 (SPSS, Chicago, IL, USA) and GraphPad Prism 5.0 (GraphPad Software, La Jolla, CA, USA) were responsible for all the statistical analyses. The quantitative data [mean ± standard deviation (SD)] were compared with Student's *t* test, and the categorical data were compared utilizing chi‐square test. Comparisons among ≥3 groups were implemented with usage of analysis of variance (ANOVA). Spearman linear correlation analysis was adopted to determine the relevant association of H19 expression with miR‐130a‐3p or miR‐17‐5p expression. Log‐rank test was implemented to analyze the tendency of cardiac cancer patients’ overall survival (OS), and Cox‐regression models were established to conduct univariate and multivariate analyses. It was believed as statistically meaningful when *P* value was less than 0.05.

## 3 RESULTS

### 3.1 H19, miR‐130a‐3p, and miR‐17‐5p expressions were compared between cardiac cancer tissues and para‐carcinoma tissues

**Figure 1:**
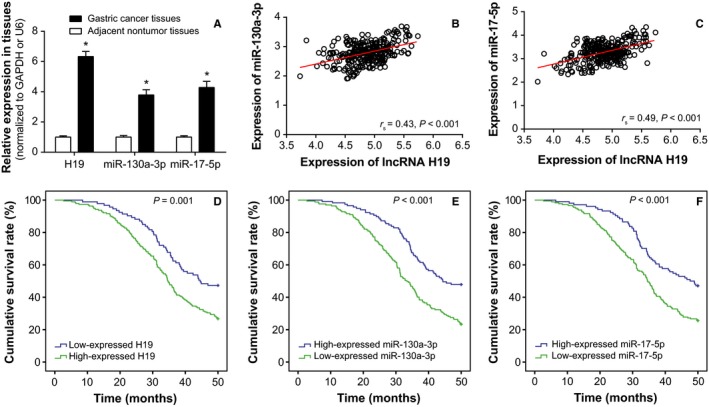
Expressions of lncRNA H19, miR‐130a‐3p, and miR‐17‐5p within cardiac cancer tissues. A, H19, miR‐130a‐3p, and miR‐17‐5p expressions were compared between cardiac cancer tissues and adjacent normal tissues. * Student's *t* test: *P* < 0.05 when compared with adjacent normal tissues. B, H19 expression was positively correlated with miR‐130a‐3p expression among the included cardiac cancer tissues. * Spearman correlation test: *r*_s_ = 0.43, *P* < 0.001. C, H19 expression was positively correlated with miR‐17‐5p expression within cardiac cancer tissues. *Spearman correlation test: *r*_s_ = 0.49, *P* < 0.001. D, Highly expressed H19 was associated with lower overall survival rate of cardiac cancer patients, when compared with lowly expressed H19. *Log‐rank test: *P* = 0.001. E, Overexpression of miR‐130a‐3p was correlated with poorer prognosis of cardiac cancer patients, when compared with underexpressed miR‐130a‐3p. *Log‐rank test: *P* < 0.001. F, MiR‐17‐5p was more expressed within cardiac cancer patients with poorer prognosis than ones within more favorable prognosis. *Log‐rank test: *P* < 0.001

According to Figure 1A, H19, miR‐130a‐3p, and miR‐17‐5p expressions were significantly up‐regulated within cancerous tissues, when compared with matched noncancerous tissues (*P* < 0.05). Besides, the included 284 cardiac cancer tissues were, respectively, divided into highly expressed H19 group (>median expression, n = 191) and lowly expressed H19 group (≤median expression, n = 93), highly expressed miR‐130a‐3p group (>median expression, n = 167) and lowly expressed miR‐130a‐3p group (≤median expression, n = 117), as well as highly expressed miR‐17‐5p group (>median expression, n = 180) and lowly expressed miR‐17‐5p group (≤median expression, n = 104; Table 2). The univariate analyses displayed that higher H19, miR‐130a‐3p, and miR‐17‐5p expressions were more frequently observed within subjects that were featured by bigger tumor size (>5 cm), invasive serous layer, metastatic lymph nodes, and III+IV stages of TNM (*P* < 0.05; Table 2). In terms of multivariate analyses, higher H19 (HR = 1.96, 95% CI: 1.01‐3.85, *P* = 0.048), miR‐130a‐3p (HR = 2.44, 95% CI: 1.35‐4.35, *P* = 0.003) or miR‐17‐5p expression (HR = 2.94, 95% CI: 1.54‐5.56, *P* = 0.001), larger tumor size (HR = 2.21, 95%CI: 1.19‐4.11, *P* = 0.012), invasive serous layer (HR = 2.67, 95%CI: 1.38‐5.16, *P* = 0.004), metastatic lymph node (HR = 2.26, 95%CI: 1.12‐4.58, *P* = 0.024) and TNM III+IV stage (HR = 3.22, 95%CI: 1.61‐6.45, *P* = 0.001) could predict the poor prognosis of cardiac cancer patients (Table 3, Figure 1D‐F). The Pearson correlation analysis also reflected that H19 expressions were positively correlated with either miR‐130a expression or miR‐17 expression (Figure 1B‐C).

**Table 2:** Linkage of H19, miR‐130a‐3p, and miR‐17‐5p expressions with clinical characteristics of cardiac cancer patients

Clinical characteristics (N = 284)	H19 expression			miR‐130a‐3p expression			miR‐17‐5p expression		
	Low	High	*P* value[Link]	Low	High	*P* value[Link]	Low	High	*P* value[Link]
Age									
>50	51	96		58	89		51	96	
≤50	42	95	0.469	59	78	0.537	53	84	0.485
Gender									
Male	55	121		78	98		69	107	
Female	38	70	0.493	39	69	0.173	35	73	0.248
Tumor size (cm)									
>5	33	99		45	87		40	92	
≤5	60	92	**0.010**	72	80	**0.023**	64	88	**0.040**
Differentiation degree									
Moderately differentiated	34	80		41	73		34	80	
+Well differentiated									
No differentiated+	59	111	0.390	76	94	0.142	70	100	0.052
Poorly differentiated									
Depth of infiltration									
Invasion of serous layer	75	121		74	122		63	133	
Not invasion of serous layer	18	70	**0.003**	43	45	0.079	41	47	**0.019**
Lymph node metastasis									
Yes	12	92		34	70		30	74	
No	81	99	**<0.001**	83	97	**0.027**	74	106	**0.039**
TNM stage									
III + IV	24	75		31	68		45	54	
I + II	69	116	**0.026**	86	99	**0.013**	59	126	**0.024**

Chi‐square test.

The *P* value in bold means a significant results which is less than 0.05.

**Table 3:** Impacts of H19, miR‐130a‐3p, and miR‐17‐5p expressions, as well as clinical characteristics on the survival rates of cardiac cancer patients

Clinical characteristics	Univariate analysis			Multivariate analysis		
	Hazard ratio	95% CI	*P* value[Link]	Hazard ratio	95% CI	*P* value[Link]
LncRNA H19 expression						
High vs Low expression	2.44	1.47‐4.17	**0.001**	1.96	1.01‐3.85	**0.048**
miR‐130a‐3p expression						
High vs Low expression	3.03	1.82‐5.00	**<0.001**	2.44	1.35‐4.35	**0.003**
miR‐17‐5p expression						
High vs Low expression	2.56	1.56‐4.35	**<0.001**	2.94	1.54‐5.56	**0.001**
Age						
>50 vs ≤50	0.78	0.48‐1.29	0.336	0.65	0.36‐1.17	0.15
Gender						
Male vs Female	1.21	0.73‐2.01	0.457	1.41	0.77‐2.60	0.266
Tumor size (cm)						
>5 vs ≤5	3.15	1.85‐5.35	**<0.001**	2.21	1.19‐4.11	**0.012**
Differentiation degree						
Moderately differentiated + Well differentiated vs No differentiated + Poorly differentiated	1.01	0.61‐1.67	0.973	0.97	0.52‐1.79	0.916
Depth of infiltration						
Invasion of serous layer vs not invasion of serous layer	2.65	1.57‐4.47	**<0.001**	2.67	1.38‐5.16	**0.004**
Lymph node metastasis						
Yes vs No	3.91	2.15‐7.11	**<0.001**	2.26	1.12‐4.58	**0.024**
TNM stage						
III + IV vs I + II	2.94	1.64‐5.24	**<0.001**	3.22	1.61‐6.45	**0.001**

Cox‐regression analysis.

The *P* value in bold means a significant results which is less than 0.05.

### 3.2 The responses of cardiac cancer cells with altered H19, miR‐130a‐3p, and miR‐17‐5p expressions to chemotherapy and radiotherapy

**Figure 2:**
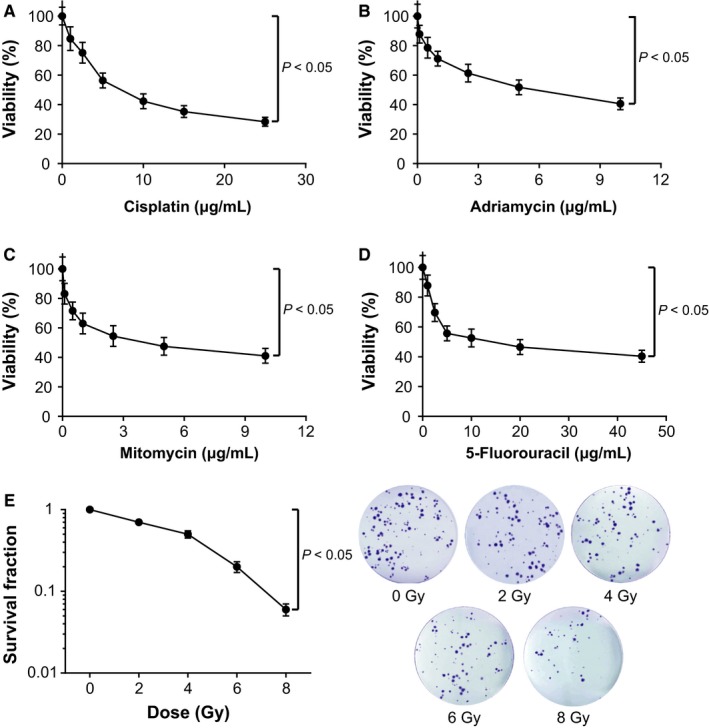
The sensitivities of cardiac cancer cells were compared regarding their responses to drugs [ie, cisplatin (A), adriamycin (B), mitomycin (C), and 5‐fluorouracil (D)] and radiotherapies (ie, 0, 2, 4, 6, and 8 Gy) (E). *Student's *t* test: *P* < 0.05

It was pointed out that the IC50 values of cardiac cancer cells were, respectively, 2.01, 8.35, 24.44, and 166.42 μg/mL under the treatments of cisplatin, adriamycin, mitomycin, and 5‐fluorouracil (Figure 2A‐D). After being subjected to radiotherapy, the cells assumed decreased survival rate with the increasing of absorbed radiation (*P* < 0.05; Figure 2E).

**Figure 3:**
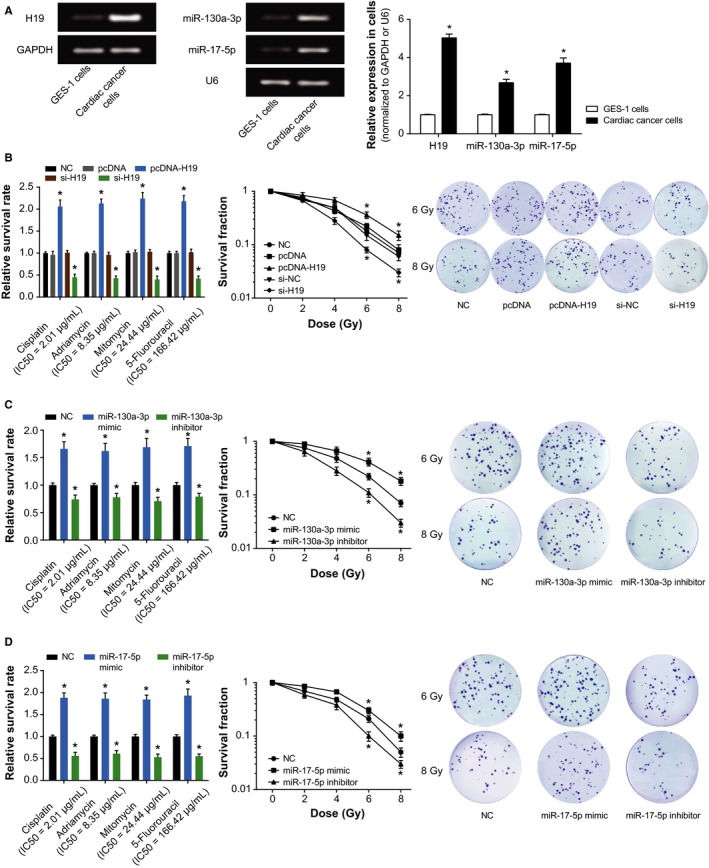
Impacts of lncRNA H19, miR‐130a‐3p, and miR‐17‐5p on the sensitivity of cardiac cancer cells to chemo‐ and radiotherapies. A, The expressions of H19, miR‐130a‐3p, and miR‐17‐5p were compared between cardiac cancer cells and normal gastric mucosal cells (ie, GES‐1). *Student's *t* test: *P* < 0.05 when compared with GES‐1. B, The effects of si‐H19 and pcDNA‐H19 on survival rates of cardiac cancer cells were evaluated after treatments with drugs (ie, cisplatin, adriamycin, mitomycin, and 5‐fluorouracil) and radiotherapy. *ANOVA test: *P* < 0.05 when compared with NC. C, The effects of miR‐130a‐3p mimic and miR‐130a‐3p inhibitor on survival rates of cardiac cancer cells were evaluated after treatments with drugs (ie, cisplatin, adriamycin, mitomycin, and 5‐fluorouracil) and radiotherapy. *ANOVA test: *P* < 0.05 when compared with NC. D, The effects of miR‐17‐5p mimic and miR‐17‐5p inhibitor on survival rates of cardiac cancer cells were evaluated after treatments with drugs (ie cisplatin, adriamycin, mitomycin, and 5‐fluorouracil) and radiotherapy. *ANOVA test: *P* < 0.05 when compared with NC. NC, negative control

More than that, H19‐siRNA4 (Figure S1A) with the largest interfering efficiency was designated as si‐H19 for this article. Also, miR‐130a‐3p and miR‐17‐5p expressions within cardiac cancer cells were also detected after transfections of miR‐130a‐3p mimic/inhibitor or miR‐17‐5p mimic/inhibitor (Figure S1B,C). The cardiac cancer cells were examined with markedly up‐regulated H19, miR‐130a‐3p, and miR‐17‐5p expressions (*P* < 0.05; Figure 3A). After treatments with chemotherapies (ie, cisplatin, adriamycin, mitomycin, and 5‐fluorouracil) and radiotherapies (ie, 6 Gy and 8 Gy), the cardiac cancer cells transfected with each of pcDNA‐H19, miR‐130a‐3p mimic, and miR‐17‐5p mimic were observed with higher survival rates than the untreated cells (all *P* < 0.05; Figure 3B‐D). Conversely, cells with inhibited H19, miR‐130a‐3p, or miR‐17‐5p expressions exhibited lower survival rate than the untreated cells (all *P* < 0.05). Interestingly, H19 seemed to affect the survival rate of cardiac cancer cells more pronouncedly than miR‐130a‐3p or miR‐17‐5p (all *P* < 0.05).

### 3.3 Impact of H19, miR‐130a‐3p, and miR‐17‐5p on proliferation, viability, and apoptosis of cardiac cancer cells

**Figure 4:**
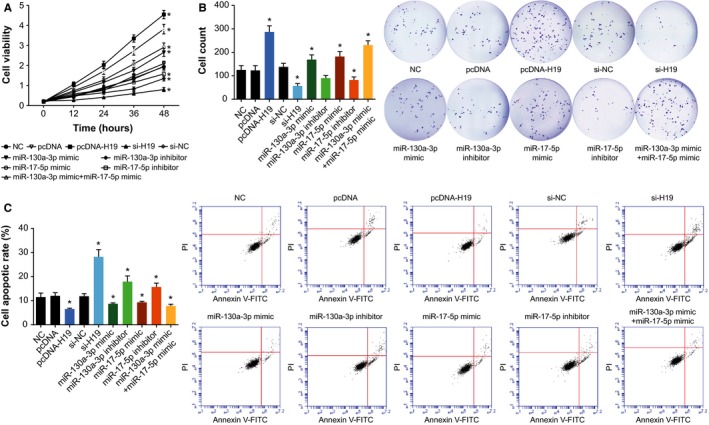
The viability (A), proliferation (B), and apoptosis (C) of cardiac cancer cells were examined among pcDNA H19, si‐H19, miR‐130a‐3p mimic, miR‐130a‐3p inhibitor, miR‐17‐5p mimic, and miR‐17‐5p inhibitor groups. *ANOVA test: *P* < 0.05 when compared with NC. NC, negative control

**Figure 5:**
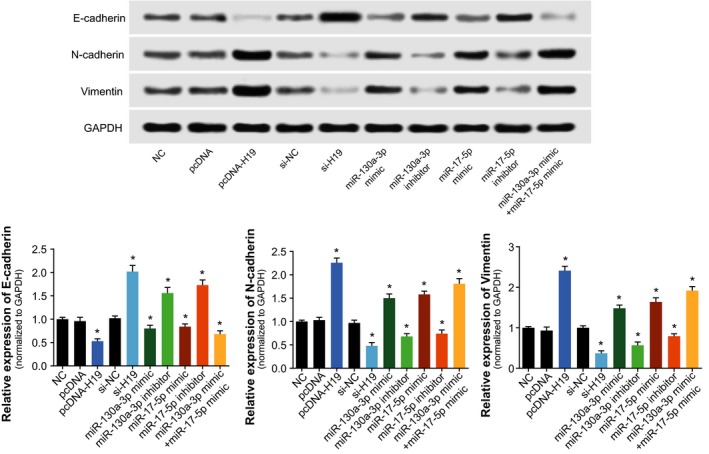
The expression levels of endothelial‐mesenchymal transition (EMT)‐specific proteins (ie, E‐cadherin, N‐cadherin, and Vimentin) were determined among pcDNA H19, si‐H19, miR‐130a‐3p mimic, miR‐130a‐3p inhibitor, miR‐17‐5p mimic and miR‐17‐5p inhibitor groups within cardiac cancer cells. *ANOVA test: *P* < 0.05 when compared with NC. NC: negative control

When compared with the NC group, the cardiac cancer cells transfected with pcDNA‐H19, miR‐130a‐3p mimic, and miR‐17‐5p mimic groups all displayed remarkably elevated viability and proliferative capacity, along with dropped percentages of apoptotic cells (Figure 4). However, transfection with si‐H19, miR‐130a‐3p inhibitor or miR‐17‐5p inhibitor all contributed to attenuated viability and proliferation, as well as facilitated apoptosis of cardiac cancer cells (Figure 4). Furthermore, transfection with pcDNA‐H19, miR‐130a‐3p mimic and miR‐17‐5p mimic also noticeably reduced E‐cadherin expression, yet fantastically picked up N‐cadherin and vimentin expressions within cardiac cancer cells (all *P* < 0.05; Figure 5). When si‐H19, miR‐130a‐3p inhibitor, or miR‐17‐5p inhibitor were put into use, completely opposite results were obtained for the cardiac cancer cells (all *P* < 0.05; Figure 5). The above results suggested that H19, miR‐130a‐3p, and miR‐17‐5p all pushed forward proliferation of cardia cancer cells and held back their apoptosis.

### 3.4 The targeted relationship between H19 and miR‐130a‐3p/miR‐17‐5p

**Figure 6:**
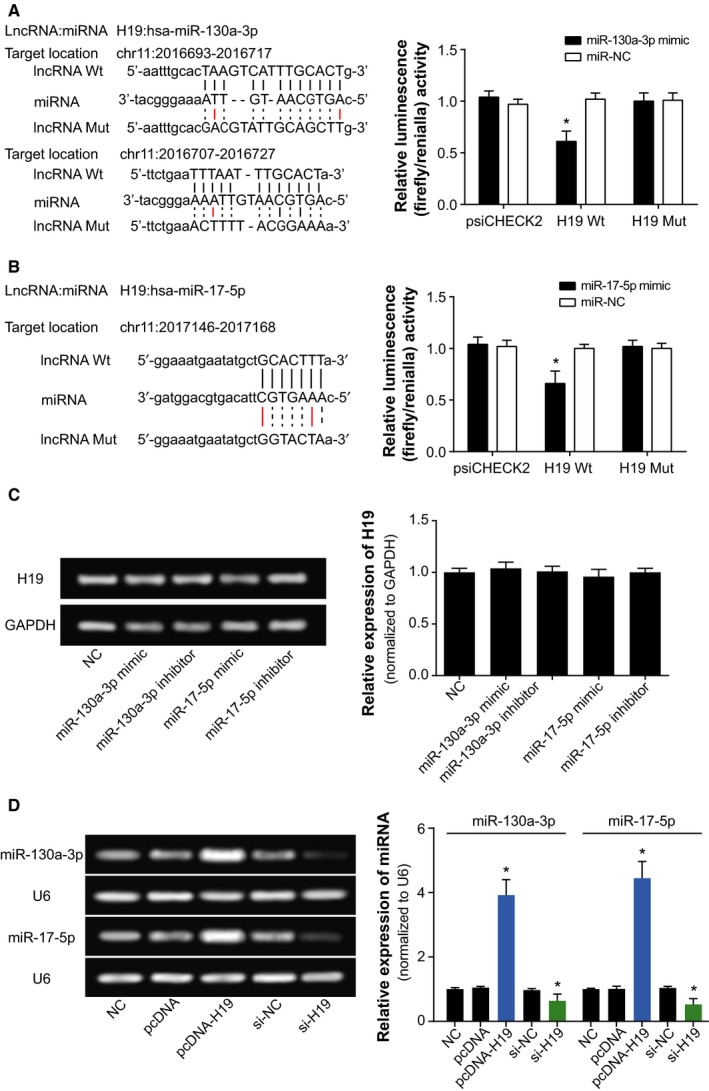
The correlations between H19 and miR‐130a‐3p or miR‐17‐5p within cardiac cancer cells. A, The targeted sites of H19 and miR‐130a‐3p were labeled, and the luciferase activities of cardiac cancer cells were evaluated after transfection of miR‐130a‐3p mimic and H19 Wt or H19 Mut.*ANOVA test: *P* < 0.05 when compared with miR‐NC+H19 Wt group. B, H19 bound to miR‐17‐5p in certain sites, and the luciferase activities of cardiac cancer cells were evaluated after transfection of miR‐17‐5p mimic and H19 Wt or H19 Mut.*Student's *t* test: *P* < 0.05 when compared with miR‐NC+H19 Wt group. C, The H19 expression was evaluated after transfection of miR‐130a‐3p mimic, miR‐130a‐3p inhibitor, miR‐17‐5p mimic or miR‐17‐5p inhibitor. *ANOVA test: *P* < 0.05 when compared with NC. D, The miR‐130a‐3p and miR‐17‐5p expressions were assessed after transfection of pcDNA‐H19 or si‐H19. *ANOVA test: *P* < 0.05 when compared with NC. NC, negative control

Dual luciferase reporter gene assays pointed out that the miR‐130a‐3p+H19 Wt group produced lower luciferase activity than miR‐130a‐3p+H19 MutT1 and miR‐130a‐3p+NC groups (*P* < 0.05), and the latter two shared similar luciferase activities (*P* > 0.05; Figure 6A). By the same logic, miR‐17‐5p+H19 Wt group displayed reduced luciferase activities in comparison with miR‐17‐5p+H19 Mut1 and miR‐17‐5p+NC groups (*P* < 0.05; Figure 6B). In addition, qRT‐PCR results mirrored that highly expressed H19 within cardiac cancer cells could remarkably raise the expressions of miR‐130a‐3p and miR‐17‐5p (*P* < 0.05; Figure 6C‐D). The above results indicated that miR‐130a‐3p and miR‐17‐5p expressions were modified after being targeted by H19.

### 3.5 Effects of H19, miR‐130a‐3p, and miR‐17‐5p on tumor formation within mice models

**Figure 7:**
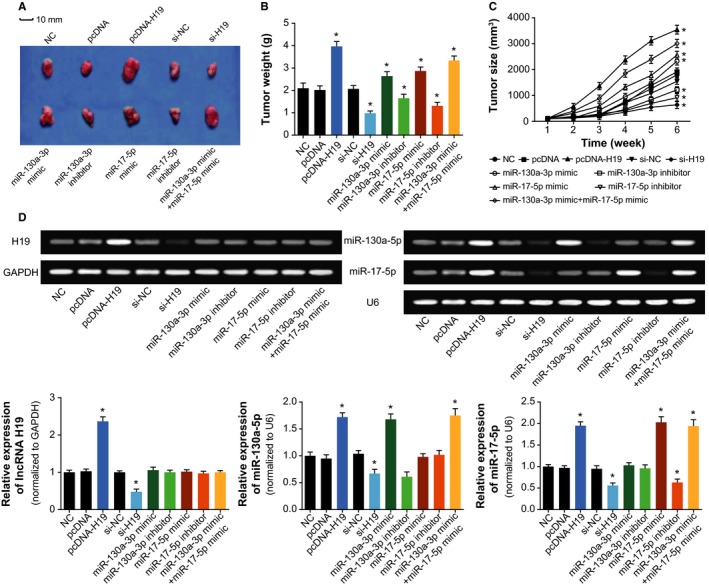
Effects of H19, miR‐130a‐3p, and miR‐17‐5p on tumor formation within mice models of cardiac cancer. A, The tumor forms were shown among mice models of pcDNA H19 (n = 10), si‐H19 (n = 10), miR‐130a‐3p mimic (n = 10), miR‐130a‐3p inhibitor (n = 10), miR‐17‐5p mimic (n = 10), and miR‐17‐5p inhibitor (n = 10) groups; B, The tumor size of mice models were compared among pcDNA H19, si‐H19, miR‐130a‐3p mimic, miR‐130a‐3p inhibitor, miR‐17‐5p mimic, and miR‐17‐5p inhibitor groups. *ANOVA test: *P* < 0.05 when compared with NC; C, The tumor weight of mice models were compared among pcDNA H19, si‐H19, miR‐130a‐3p mimic, miR‐130a‐3p inhibitor, miR‐17‐5p mimic, and miR‐17‐5p inhibitor groups. *ANOVA test: *P* < 0.05 when compared with NC; D, Expressions of H19, miR‐130a‐3p, and miR‐17‐5p were determined within tissues of mice models in the pcDNA H19, si‐H19, miR‐130a‐3p mimic, miR‐130a‐3p inhibitor, miR‐17‐5p mimic, and miR‐17‐5p inhibitor groups. *ANOVA test: *P* < 0.05 when compared with NC. NC, negative control

In line with Figure 7, the tumor size and weight of pcDNA‐H19, miR‐130a‐3p mimic and miR‐17‐5p mimic groups were significantly above those of control group (*P < *0.05), whereas depression of H19, miR‐130a‐3p, and miR‐17‐5p expressions rendered the tumor size and weight of mice models to be hypo‐control (*P < *0.05). Moreover, though miR‐130a‐3p+miR‐17‐5p group generated bigger tumor size and weight than either miR‐130a‐3p or miR‐17‐5p group (*P < *0.05), its tumor size and weight were still smaller than the pcDNA‐H19 group (*P < *0.05). In addition, the up‐regulated H19 expression could be detected within mice of pcDNA‐H19 group (*P < *0.05), and the pcDNA‐H19‐treated mice exhibited dramatically boosted expressions of miR‐130a‐3p and miR‐17‐5p (*P < *0.05).

## 4 DISCUSSION

Since cardiac cancer was easily confused with peptic ulcer, and symptomatic treatment could generally relieve its clinical manifestations, crowds of cardiac cancer patients refused to receive further examinations. In addition, the complex anatomy of cardiac area enabled the cavity shape to be irregular, making it tough to detect small concavities of abdomen.^29^, ^30^ Thus, confirmed diagnoses of cardiac cancer were prone to be delayed, and pointed treatment for cardiac cancer became intractable. Furthermore, although chemo‐/radiotherapies have been developed for improving survival of cardiac cancer patients, the patients gradually displayed an enhancive resistance to the therapies, which were assumed as facilitated metastasis and relapse of neoplasms. Hence, further elucidating the molecular mechanisms related with cardiac cancer cells’ chemo‐ and radio‐sensitivity appeared crucial for adequate treatment of cardiac cancer.

Instead of being the transcriptional noise, it was demonstrated that lncRNAs could interact with nucleic acids or proteins, so as to regulate genetic expressions that were involved with drug resistance of tumor cells.^31^ As a matter of fact, a portion of chemo‐therapeutic agents, including cisplatin, adriamycin, mitomycin, and 5‐fluorouracil,^32^, ^33^, ^34^ were designed to inhibit tumor growth by inducing apoptosis of tumor cells, so deregulation of cell apoptosis could initiate drug resistance.^35^ The inhibition of cell apoptosis, activation of cell survival, and regulation of cell cycle were also identified as mechanisms underlying raised radio‐resistance of malignancies.^36^, ^37^, ^38^, ^39^, ^40^, ^41^, ^42^, ^43^, ^44^

Intriguingly, the H19 investigated here was suggested as an obstacle to efficaciously treating gastric cancer due to its responsibility for drug tolerance.^45^ Its expression within adriamycin‐resistant gastric cancer cell line (ie, R‐HepG2) was nearly seven times more than that within HepG2 cell line that was relatively sensitive to adriamycin.^46^ Moreover, previous documentations also revealed a positive relationship between H19 expression and progression of breast cancer,^47^ liver cancer,^48^ lung cancer,^49^ cervical cancer,^50^ and gastric cancer.^51^ The contributions of H19 to promoting neoplastic progression and chemo‐/radio‐resistance seemed to be well applicable to the cardiac cancer explored within this study (Figure 2). Besides, since up‐regulated H19 expression rendered cardiac cancer patients with enlarged risk of unfavorable overall survival (Figure 1), it was insinuated that H19 could interfere with the recovery of cardiac cancer patients by depressing apoptosis and expediting growth of cancer cells (Figures 3 and 4). Furthermore, H19 was hypothesized to additionally regulate expressions of EMT‐specific biomarkers (Figure 5), which also lowered sensitivity of cardiac cancer cells to chemotherapies.^52^ The EMT process robbed cells of their polarity and intercellular adhesion, which made for the occurrence of drug resistance.^53^ Besides, it was increasingly held that EMT was closely linked with the biological phenotype of tumor stem cells, which were verified as cell populations that were strongly resistant to drugs.^54^

Within this investigation, we attempted to symbolize EMT process with certain biomarkers, namely, E‐cadherin, N‐cadherin, and vimentin (Figure 5). The E‐cadherin investigated here was a Ca2+‐dependent transmembrane glycoprotein, and it could lead to weakened adhesion among tumor cells.^55^ Another membrane protein, N‐cadherin, was reported to lessen the inhibitory effects of E‐cadherin on tumor metastasis through combining with p120‐ctn to boost internalized degradation of E‐cadherin.^56^, ^57^ Besides, the vimentin therein was a cytoskeleton protein, and it probably contributed to promoted tumor migration and invasion by modifying the form of E‐cadherin and β‐catenin.^58^, ^59^, ^60^ Nonetheless, relevant migratory and invasive experiments that visually displayed the EMT process of cells were still demanded to confirm this hypothesis.

As for how H19 achieved the effects as mentioned above, a theory of competing endogenous RNAs (ceRNAs) proposed that lncRNA, pseudo‐RNA, or circRNA could competitively combine with miRNAs to modify onset of neoplastic malignancies.^35^, ^61^, ^62^, ^63^ The miRNAs have been considered as the major determinant of neoplastic progression and chemo‐/radio‐resistance of tumors.^64^, ^65^, ^66^, ^67^ As presented in Figure 6, the luciferase reporter gene assay conducted revealed that H19 could sponge miR‐130a‐3p and miR‐17‐5p, and alter their expressions. Among them, the miR‐17‐5p belonged to polycistronic miR‐17‐92 gene cluster,^68^ and it was highly expressed within gastric cancer,^69^, ^70^ lung cancer,^71^ blood cancer, and solid tumors.^72^ For another, miR‐130a expression was up‐regulated within basal cell carcinoma, esophageal cancer, T‐cell leukemia, and gastric cancer tissues than within normal tissues.^73^, ^74^, ^75^, ^76^, ^77^ The current investigation of cardiac cancer also validated that miR‐17‐5p and miR‐130a‐3p were both overexpressed within neoplasms, which rendered greater odds of poor prognosis among the cardiac cancer patients (Figure 1). More than that, the couple of miRNAs could participate in modulating cell apoptosis, cell viability, and expression of EMT‐specific proteins (Figures 4 and 5), which made their positive impacts on chemo‐resistance and radio‐resistance of cardiac cancer cells possible (Figures 2 and 3). It has also been documented that transfection of miR‐17‐5p inhibitor could markedly improve the chemo‐sensitivity of pancreatic cells (ie, Panc‐1 and BxPC3) to gemcitabine via reduction of cells’ spontaneous apoptosis.^78^ And down‐regulation of miR‐130a‐3p potentially induced resistance of hepatoma cells to gemcitabine, ovarian cancer cells to paclitaxel or cisplatin, non‐small‐cell lung cancer cells to gefitinib and prostate cancer cells to paclitaxel.^26^, ^79^, ^80^, ^81^

Above all, H19 targeting miR‐130a‐3p and miR‐17‐5p could encourage radio‐ and chemo‐resistance of cardiac cancer cells, which might be conducive to improving the treatment efficacy for cardiac cancer. Nonetheless, several limitations still existed, and further explorations were recommended. Firstly, it demanded to be figured out that whether there presented a loop among H19, miR‐130a‐3p, miR‐17‐5p, and other molecules. Secondly, the included samples were limited in its size and constitution, and a larger scale of cardiac cancer population needed to be gathered. Thirdly, the cardiac cancer patients we included were not arranged for stratified analyses. Finally, ATP‐binding cassette (ABC) was a vital drug transporter that affected multidrug resistance (MDR),^82^ so the interactions of H19, miR‐130a‐3p, and miR‐17‐5p with ABC awaited further proof.

## CONFLICT OF INTEREST

None declared.
