# Supplementary material for: LncRNA H19 interacted with miR‐130a‐3p and miR‐17‐5p to modify radio‐resistance and chemo‐sensitivity of cardiac carcinoma cells
Source: Cancer Med. 2019 Mar 6;8(4):1604–18. doi: 10.1002/cam4.1860 (PMC6488143; doi:10.1002/cam4.1860)
Supplement: Supplementary file 1 [file CAM4-8-1604-s001.pdf]

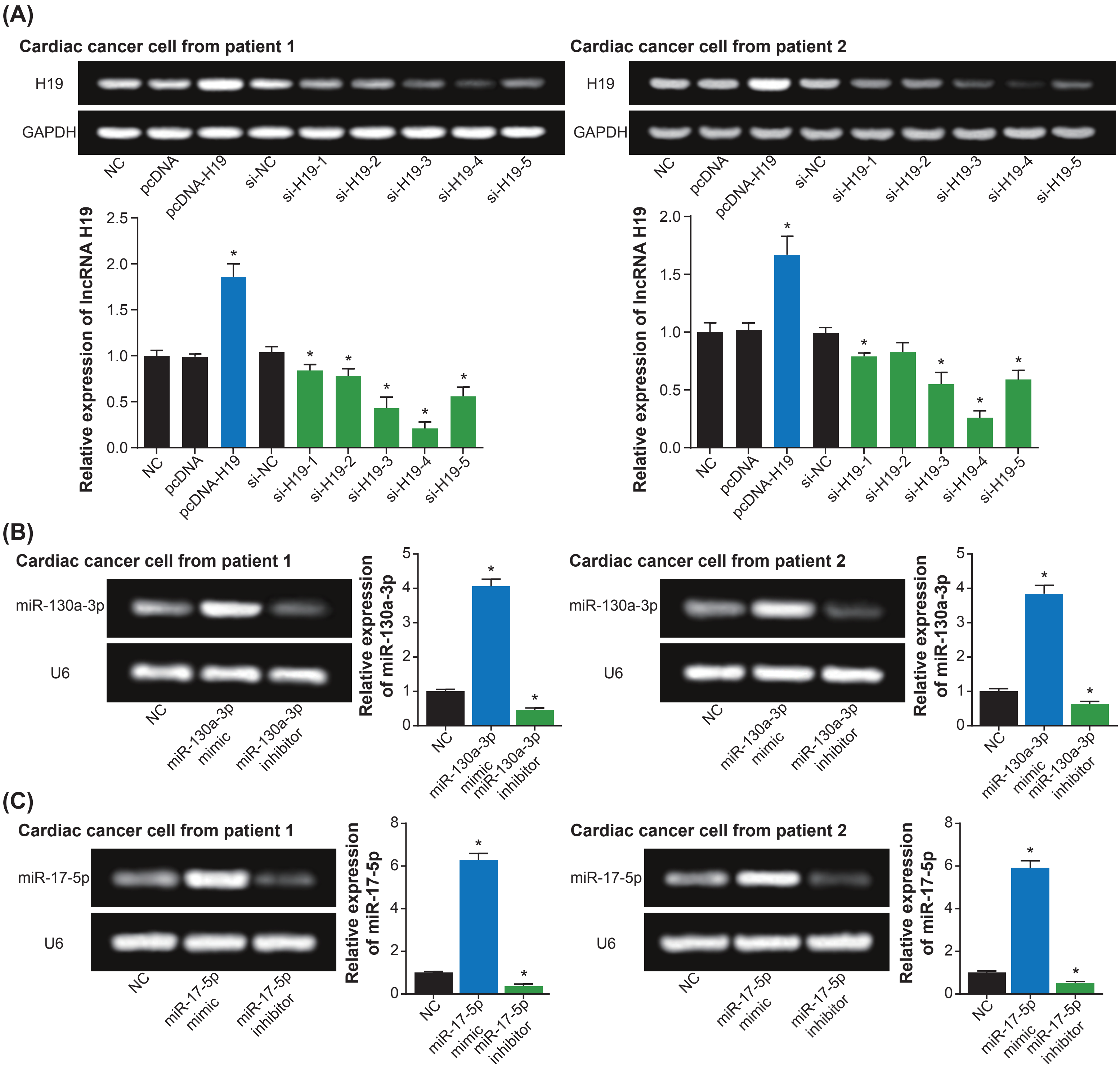
(A)
Cardiac cancer cell from patient 1
H19
GAPDH
NC
pcDNA
pcDNA-H19
si-NC
si-H19-1
si-H19-2
si-H19-3
si-H19-4
si-H19-5
Relative expression of lncRNA H19
Cardiac cancer cell from patient 2
(B)
Cardiac cancer cell from patient 1
miR-130a-3p
U6
miR-130a-3p mimic
miR-130a-3p inhibitor
Relative expression of miR-130a-3p
Cardiac cancer cell from patient 2
(C)
Cardiac cancer cell from patient 1
miR-17-5p
U6
miR-17-5p mimic
miR-17-5p inhibitor
Relative expression of miR-17-5p
Cardiac cancer cell from patient 2
