# Supplementary material for: LncRNA H19 interacted with miR‐130a‐3p and miR‐17‐5p to modify radio‐resistance and chemo‐sensitivity of cardiac carcinoma cells
Source: Cancer Med. 2019 Mar 6;8(4):1604–18. doi: 10.1002/cam4.1860 (PMC6488143; doi:10.1002/cam4.1860)
Supplement: Supplementary file 2 [file CAM4-8-1604-s002.docx]

**Supplementary table 1 The sequences of lncRNA H19-siRNA.**

| **SiRNA** | **Target sequence**  **21 nt target+2 nt overhang** | **Sequences (5’→3’)** | **Target position** |
| --- | --- | --- | --- |
| H19-siRNA1 | CAGAAGAATGGTACAAATCCAAG | 5’-TGGATTTGTACCATTCTTCTG-3’ (sense)  5’-GAAGAATGGTACAAATCCAAG-3’(antisense) | 24-46 |
| H19-siRNA2 | CCCTTAAAGGAACCAATGAGTCC | 5’-ACTCATTGGTTCCTTTAAGGG-3’ (sense)  5’-CTTAAAGGAACCAATGAGTCC-3’ (antisense) | 159-181 |
| H19-siRNA3 | AGGATGAGATTCAGAATATGAAG | 5’-TCATATTCTGAATCTCATCCT-3’ (sense)  5’-GATGAGATTCAGAATATGAAG-3’ (antisense) | 261-283 |
| H19-siRNA4 | CTGGTTGATACCCACTCAAAAAG | 5’-TTTTGAGTGGGTATCAACCA-3’ (sense)  5’-GGTTGATACCCACTCAAAAAG-3’ (antisense) | 461-483 |
| H19-siRNA5 | GACAGGTTATCAACGAAACTTCT | 5’-AGAAGTTTCGTTGATAACCTG-3’ (sense)  5’-CAGGTTATCAACGAAACTTCT-3’ (antisense) | 519-541 |
| SiRNA-NC |  | 5’-TGGCTAAGGAGAGTAGTCTGA-3’ (sense)  5’-TCAGACTACTCTCCTTAGCCA-3’ (antisense) | -- |

NC: negative control.
